# The Influence of Melatonin and Light on VEGF Secretion in Primary RPE Cells

**DOI:** 10.3390/biom11010114

**Published:** 2021-01-16

**Authors:** Alexa Klettner, Miriam Kampers, Daniela Töbelmann, Johann Roider, Manuela Dittmar

**Affiliations:** 1 Department of Ophthalmology, Campus Kiel, University Medical Center Schleswig-Holstein (UKSH), 24105 Kiel, Germany; miriam.kampers@web.de (M.K.); johann.roider@uksh.de (J.R.); 2 Department of Human Biology, Zoological Institute, University of Kiel, 24118 Kiel, Germany; dtoebelmann@zoologie.uni-kiel.de (D.T.); mdittmar@zoologie.uni-kiel.de (M.D.)

**Keywords:** vascular endothelial growth factor (VEGF), retinal pigment epithelium (RPE), melatonin, diurnal rhythm, BMAL1, light induction

## Abstract

(1) Background: Retinal pigment epithelial cells (RPE) cells constitutively secrete vascular endothelial growth factor (VEGF) in the retina, protecting the neuronal cells and the choroid. Increased VEGF secretion, however, can result in neovascularization and edema. Many factors regulate VEGF secretion. In this study, we investigated the effect of external stimuli in relation to diurnal rhythm on constitutive VEGF secretion. (2) Methods: Single-eye RPE cell culture was prepared from porcine eyes. RPE cells were cultured in darkness, treated with daylight or room light, and treated with melatonin at different time frames, either respectively or in combination. Supernatants were collected and VEGF content evaluated using ELISA. Expression of the clock protein BMAL1 was evaluated with Western blot. (3) Results: VEGF secretion of the RPE shows a diurnal rhythm. While the rhythm is not influenced by either light or melatonin, the amount of secreted VEGF can be increased by nocturnal melatonin, especially in combination with morning daylight. These findings disclose another layer of VEGF regulation in the retina.

## 1. Introduction

The retinal pigment epithelium (RPE) is an epithelial monolayer situated beneath the neuroretina. It is in close contact and interaction with the photoreceptors and the underlying choroid [1]. The RPE has a plethora of functions in the retina, including phagocytosis of photoreceptor outer segments, recycling the visual pigment, retinal waste disposal, and constituting the outer blood retinal barrier [2]. In addition, the RPE is a major source of the vascular endothelial growth factor (VEGF) in the retina [3,4,5,6]. VEGF is notorious for its importance in pathological alterations in the retina, inducing neovascularizations and edema in diseases like exudative age-related macular degeneration (AMD), diabetic retinopathy, or retinopathy of prematurity [7,8,9]. However, VEGF has many protective functions in the retina [6]. It protects retinal neurons and the RPE, as well as endothelial cells, and keeps up the fenestration of the endothelial cells of the choroid [3,4,10,11,12], which is important for the nutrient supply of the photoreceptors [1]. Constitutive VEGF expression and secretion in the RPE is regulated by many factors [13,14], which may differ from the regulation of VEGF induced by pathological stimuli such as hypoxia, oxidative stress, inflammatory signaling, and other [8,15,16,17,18,19]. Indeed, the expression of VEGF is regulated at many levels in order to uphold the appropriate amount for protection and oxygen supply [20,21,22,23]. 

Recently, a potential influence of circadian regulation on VEGF expression has been described in systems outside the retina. In the angiogenesis model of the developing zebrafish, it was shown that circadian oscillation controls developmental angiogenesis, with the circadian regulator BMAL1 targeting the promoter region of the VEGF gene, and a circadian rhythm of expression of VEGF in the developing zebrafish was observed [24]. In the promoter regions of the VEGF gene, several potential BMAL1-binding E-boxes were found [24]. In a mouse model of transplanted tumors, circadian variations of the VEGF levels in Lewis lung carcinoma and sarcoma180 were found, which correlated with the efficiency of photodynamic therapy (PDT) treatment in these cells [25]. Also, it was shown that the levels of VEGF mRNA in tumors in response to hypoxia fluctuated in a circadian fashion [26]. In addition, patients with POEMS (polyneuropathy, organomegaly, endocrinopathy, m-protein skin changes) syndrome exhibit circadian oscillation of the VEGF plasma level, with a peak at night and a decrease during daytime [27]. 

Conversely, the adult retina is a post-developmental tissue with no active angiogenesis, as opposed to developmental and tumorous tissues. However, as noted above, the retina constitutively secretes VEGF and, moreover, is a tissue with an inherent circadian rhythm [28,29]. Neurons of the inner retinal layers are capable of generating a circadian rhythm and express clock genes (Bmal1, Clock, Per1/2, Cry1/2) [30]. The most important molecules in entraining the circadian rhythm with the day-night rhythm (light-dark rhythm) in the retina are melatonin and dopamine [31]. In addition, the RPE in situ exhibits a circadian behavior. For example, phagocytosis of the photoreceptor outer segment by the RPE is conducted in a diurnal manner. In rodents, it has been described to occur 1-2 h after light onset [32,33]. Of note, the timing differs in vitro, where phagocytosis is predominantly happening during dark intervals and becomes reduced by continuous light exposure [34]. In addition, autophagy in the RPE has also been shown to exhibit a circadian rhythm that is independent of light conditions [35]. The circadian clock of the RPE has been shown to also be independent from central input by the suprachiasmatic nuclei (SCN) in the hypothalamus of the brain, which is considered to be the body’s pacemaker [36]. To be entrained to the light-dark cycle, RPE cells need retinal input, which has been supposed to be communicated via humoral signals, such as melatonin [31]. In addition, in murine RPE/choroidal explants, RPE cells were shown to exhibit an independent circadian rhythm of PER2 expression when cultured without retinal contact, which is not dependent on light exposure [37]. Melatonin has been implicated in entraining the circadian rhythm in the eye and the timing of diurnal activities in the RPE [38,39]. In addition, it protects RPE cells from oxidative stress and has been suggested to exert protection against the development of age-related macular degeneration [40,41,42]. Also, there have been indications that melatonin can regulate VEGF, e.g., by reducing hypoxia-induced, HIF-1-dependent VEGF upregulation [43]. The retina is constantly exposed to visible light, and such exposure can also have an influence on VEGF secretion, for instance, it induces VEGF in retinoblastoma cells via a pathway including the transcription factor Sp1 [44].

In this study, we aimed to investigate whether VEGF secretion in the RPE was related to a circadian rhythm in vitro. For this, we investigated single-eye RPE cultures regarding their VEGF secretion in relation to time, light stimuli, and melatonin.

## 2. Materials and Methods

### 2.1. Primary Single-Eye RPE Cell Culture

**Figure 1:**
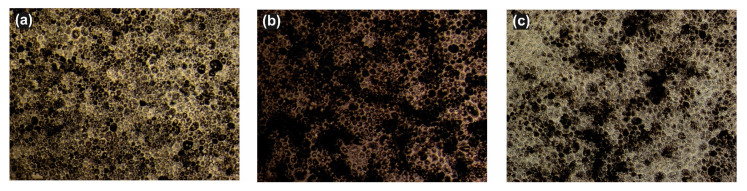
Exemplary pictures of single-eye retinal pigment epithelium (RPE) cell culture at confluence. All cultures used for experimentation were confluent and exhibited a typical cobblestone-like morphology. Note the variation in the pigmentation of the RPE cells. (**a**) Medium pigmentation; (**b**) dark pigmentation, (**c**) patchy pigmentation. Magnification × 100.

For all cell culture experiments, single-eye cell cultures were used. For this, RPE cell culture was prepared as previously described [45,46], with alterations. In brief, porcine eyes were obtained from a local slaughter house, cleaned of adjacent tissue, and the cornea, lens, vitreous, and retina were removed. RPE cells were dispatched by trypsin digestion and cultivated in Dulbecco’s modified Eagle’s medium (DMEM, PAA) supplemented with penicillin/streptomycin (1%), l-glutamine, amphotericine B (0.5 μg/mL), HEPES (25 mM), sodium-pyruvate (110 mg/mL) (all PAA), and 10% fetal calf serum (Linaris, Wertheim-Bettingen, Germany). Cells from one single eye were seeded in 1 well of a 12 well plate to collect the supernatant at the different time points or in 4 wells of a 24-well plate to collect lysates after respective treatment. Cells were grown to confluence (2–3 weeks) before experimentation. Morphology was assessed with light microscopy (Figure 1). Cells were kept in the dark during cultivation, and involuntary light exposure was avoided by the use of aluminum foil. Light exposure of the cells was done according to experiments. No entrainment was conducted apart from the light impulses and/or melatonin treatment as described below.

### 2.2. Cell Treatment

#### 2.2.1. Light Impulse

Cells were treated either with room light (Thermo Scientific Safe 2010, 710 Lux, measured with a PCE-172, PCE Group, Meschede, Germany) or daylight (light therapy device Vitality, MedNovis, no. 13050, Davita Company, Denver, CO, USA: 5000 Lux (distance 26 cm)) for 20 min at indicated time points (morning: 7:40–8:00; evening 19:40–20:00). We used these two types of light in order to compare the influence of light usually found in laboratory settings (room light) and light of biological relevance for the RPE cells (daylight). We limited the treatment to 20 min to avoid toxic effects of the illumination [47].

#### 2.2.2. Treatment with Melatonin

Cells were treated with melatonin (Sigma, Deisenhofen, Germany, 100 µM [48,49]) at indicated time points (20:00–8:00). Melatonin was dissolved in ethanol. Respective volumes of ethanol were applied to controls.

#### 2.2.3. Combined Treatment with Light and Melatonin

**Figure 2:**
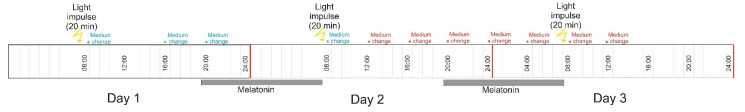
Schematic of single-eye RPE cell culture treatment with light and melatonin experimental set-up. Light impulse was given every day between 07:40 – 08:00 (yellow arrows). The medium was exchanged at time points indicated with *, blue indicating medium exchange only, red indicating medium change with supernatant collection. Melatonin (or an appropriate volume of the solvent ethanol) was added in the time between 20:00 and 08:00 (indicated by grey bar). No melatonin was added between 08:00 and 20:00.

In the experiments investigating the combined effect of light and melatonin, four different cell treatments were compared: cells kept in complete darkness (control, D), cells treated with daylight impulse (L), cells kept in darkness treated with melatonin (MD), cells treated with daylight impulse and melatonin (ML). On day 1 of the experiment, at 07:40–08:00, L and ML were exposed to the light impulse. At 16:00, the medium was exchanged. At 20:00, the medium was exchanged, and melatonin (dissolved in ethanol) was added to MD and ML, while the respective volume of ethanol was added to D and L. On day 2, L and ML were treated with the daylight impulse again at 7:40 to 8:00. At 08:00, the medium was replaced with fresh medium without melatonin (or ethanol). At 12:00 and 16:00, the medium was collected and exchanged with medium without melatonin (or ethanol). At 20:00, 24:00, and 04:00, the medium was collected and exchanged with medium containing melatonin (or ethanol). On day 3, the light impulse was given to L and ML at 07:40–08:00. At 08:00 and 12:00, the medium was collected and exchanged with medium without melatonin (or ethanol). A schematic of the experimental set-up is presented in Figure 2.

### 2.3. ELISA

Supernatants were harvested from the single-eye cell culture at 12:00, 16:00, 20:00, 24:00, 8:00, and 12:00 (following day; 24 h after collecting of the first sample) by collection and centrifugation. VEGF content was analysed and measured with DuoSet VEGF-A ELISA (R&D Systems, Wiesbaden, Germany). ELISAs were conducted according to the manufacturer’s instructions.

### 2.4. Western Blot

Protein concentration was measured using the Bradford assay (Roth, Karlsuhe, Germany). Samples of 15 µg each were heated for 5 min at 95 °C in sodium dodecyl sulfate (SDS) sample buffer (Merck, Darmstadt, Germany), separated by 12% SDS-polyacrylamide gel electrophoresis (SDS-PAGE), electro-blotted onto nitrocellulose membranes (Roth, Karlsruhe, Germany or Bio-Rad, Hercules, CA, USA), and washed three times for 5 min each in tris-buffered saline with 0.1% Tween-20 (TBST). Membranes were blocked in 5% nonfat dry milk (Roth, Karlsruhe, Germany) in TBST for 1 h at room temperature. Membranes were subsequently probed with anti-BMAL1 rabbit monoclonal antibody (1:1000 dilution, Cell Signaling Technology, Danvers, MA, USA) diluted in blocking solution and anti-β-actin rabbit monoclonal antibody (1:4000 dilution, Cell Signaling Technology, Danvers, MA, USA) diluted in 5% Albumin Fraction V (Roth, Karlsruhe, Germany) in TBST. Following incubation with the HRP-linked anti-rabbit secondary antibody (diluted 1:2000 for BMAL1 and 1:7000 for β-Actin, Cell Signaling Technology, Danvers, MA, USA), immunoblots were developed using Clarity Max ECL Western Blot Substrate (Bio-Rad, Hercules, CA, USA) and analyzed by enhanced chemiluminescence in a ChemiDocTM Touch Imaging System with Image LabTM Touch Software version 2.5.0.07 (Bio-Rad, Hercules, CA, USA). Afterwards, Image Lab Software version 5.2.1 (Bio-Rad, Hercules, CA, USA) was used for densitometric evaluation. All blots were normalized with a HeLa Whole Cell RIPA Extract (Abcam, Cambridge, UK), and the ratios between BMAL1 protein and β-Actin protein were calculated.

### 2.5. Statistics

Statistical analyses were performed using SPSS version 22 software package for MS Windows (SPSS Inc., Chicago, IL, USA). Data were expressed as means ± standard error of the mean (SEM). Normal distribution of data was tested using the Shapiro-Wilk method. Since the data of most variables did not follow a normal distribution, the Friedman test and Kruskall-Wallis test were used for comparison of k paired samples (clock times) or k independent samples (intervention conditions: darkness, light impulse, melatonin application, and combined interventions), respectively. Comparison of two paired or two independent samples was performed using the Wilcoxon matched pairs test or Mann-Whitney U test, respectively. *p*-values < 0.050 were considered statistically significant. Cosinor analysis, adapted to a 24 h period, was used to test the circadian rhythmicity of VEGF and BMAL1 using the software “Acro” version 3.5 [50], calculating mesor (mean of the circadian oscillation; midline estimating statistic of rhythm), amplitude of the curve (half of the sinusoid), and acrophase (timing of the cosine maximum) with a 95% confidence interval (CI) and an index of goodness of fit. The index of goodness of fit was computed as the ratio of the sums of squares of the best fit and the worst fit.

## 3. Results

### 3.1. Circadian Variation

**Figure 3:**
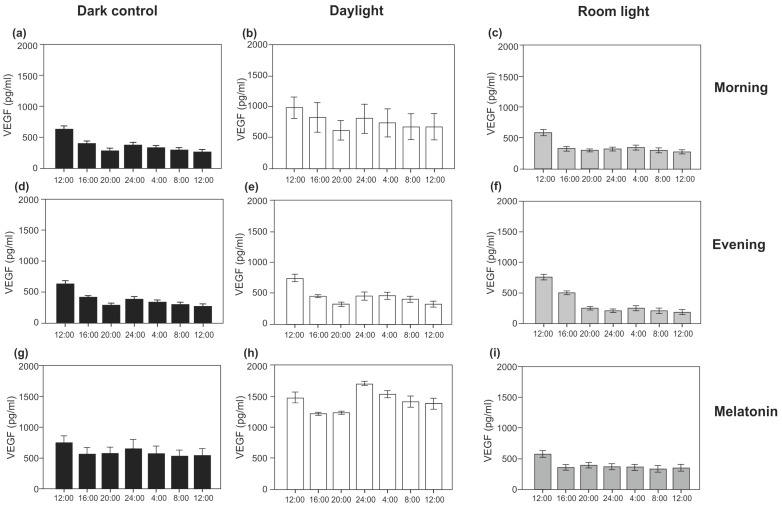
Twenty-four-hour profiles from 12:00 to 12:00 of vascular endothelial growth factor (VEGF) protein content in the supernatant from RPE cells in darkness as well as under light and melatonin intervention conditions. Values are presented as mean ± standard error of mean (SEM). Light induction: morning: 07:40–08:00, evening: 19:40–20:00; room light = 710 Lux, daylight = 5000 Lux. Melatonin induction 100 µM. (**a**) Dark control (*N* = 40), (**b**) Daylight morning (*N* = 8), (**c**) Room light morning (*N* = 18), (**d**) Dark control (*N* = 40), (**e**) Daylight evening (*N* = 5), (**f**) Room light evening (*N* = 5), (**g**) Melatonin dark (*N* = 19), (**h**) Melatonin daylight morning (*N* = 5), (**i**) Melatonin room light morning (*N* = 14). For each condition (**a**–**i**) there was a significant variation in VEGF content over the 24 h (*p* < 0.010, cf. Table 1).

The 24 h profiles for VEGF secretion into the supernatant of cultured cells were analysed for different conditions (darkness, after external light and melatonin intervention conditions). Results are presented in Figure 3 and Table 1. In constant darkness, the VEGF content showed a significant 24 h variation with an increase at around 24:00. Not only in darkness, but also in the intervention conditions, the VEGF content displayed a significant variation within 24 h, with the highest increase at around 24:00 (*p* < 0.010, Friedman test, Table 2). Thus, external melatonin and light exposure did not alter time of maximum VEGF content. Even if there was a significant 24 h variation in VEGF content, the cosinor analysis did not confirm a statistically significant circadian rhythmicity of VEGF content (*p* > 0.050) for any condition.

### 3.2. Light Induction

**Figure 4:**
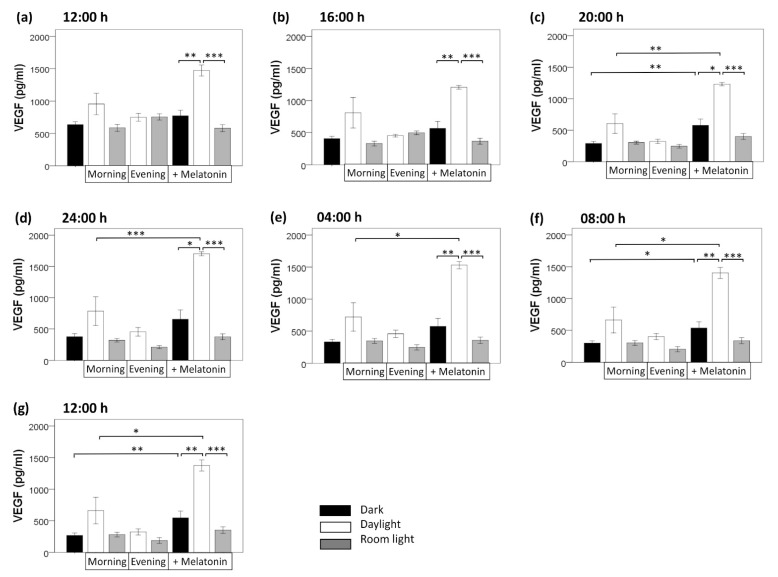
Comparison of VEGF protein content in the supernatant from RPE cells at different time points (from (**a**) 12:00 to (**g**) 12:00) in darkness as well as under light and melatonin intervention conditions. Values are presented as mean ± standard error of mean. Light induction: morning: 07:40–08:00, evening: 19:40–20:00; room light = 710 Lux, daylight = 5000 Lux. Melatonin induction 100 µM. *, *p* < 0.050; **, *p* < 0.010; ***, *p* < 0.001.

Compared with the dark condition, light induction/exposure did not significantly alter VEGF secretion, neither room light nor daylight, neither morning nor evening light induction (Figure 4). This held true for all time points (12:00, 16:00, 20:00, 24:00, 04:00, 08:00, 12:00; each, *p* > 0.050, U test). Of interest, compared with the dark condition, morning daylight induction non-significantly increased VEGF content at all clock time points (12:00, 16:00, 20:00, 24:00, 04:00, 08:00, and 12:00), while evening daylight induction did not (*p* > 0.050). Neither morning nor evening application of room light affected VEGF secretion at any clock time point (*p* > 0.050)].

### 3.3. Melatonin Treatment

By contrast, melatonin induction increased VEGF secretion in darkness at all seven time points within 24 h, being statistically significant at time point 20:00 (*p* = 0.003, U test), 08:00 (*p* = 0.024), and 12:00 (*p* = 0.008). When melatonin induction was combined with daylight (morning) induction (compared with melatonin induction in darkness), a stronger increase of VEGF secretion occurred at all seven time points (*p* = 0.002 at 12:00, *p* = 0.009 at 16:00, *p* = 0.015 at 20:00, *p* = 0.019 at 24:00, *p* = 0.007 at 04:00, *p* = 0.001 at 08:00, *p* = 0.004 at 12:00; U tests). By contrast, combining melatonin induction with room light (morning) induction did not significantly alter VEGF secretion at any clock time point (*p* > 0.050, U tests) (compared with melatonin induction in darkness) (Figure 4).

Taken together, a 24 h variation of VEGF secretion persisted in constant darkness in the absence of light as external zeitgeber. External light exposure and external melatonin stimulus did not change the 24 h variation of VEGF secretion (maximum level of VEGF secretion remained at about 24:00), but increased the production level of VEGF over 24 h. Melatonin application significantly increased VEGF secretion at 20:00, 8:00, and 12:00. Furthermore, combining external melatonin with daylight application amplified this effect leading to the strongest increase in VEGF secretion over 24 h.

### 3.4. BMAL-1 expression

BMAL1 is a clock protein involved in the regulation of the circadian rhythm in RPE cells [51]. We investigated whether external light or external melatonin changed the production level and circadian rhythm of BMAL1 protein content in primary, porcine single-eye RPE. Untreated cells were kept in constant darkness (DD condition), showing whether in the absence of light as external zeitgeber a circadian rhythmicity could be found. Also, we aimed to investigate whether melatonin may modulate oscillations of clock protein BMAL1 in the RPE.

**Figure 5:**
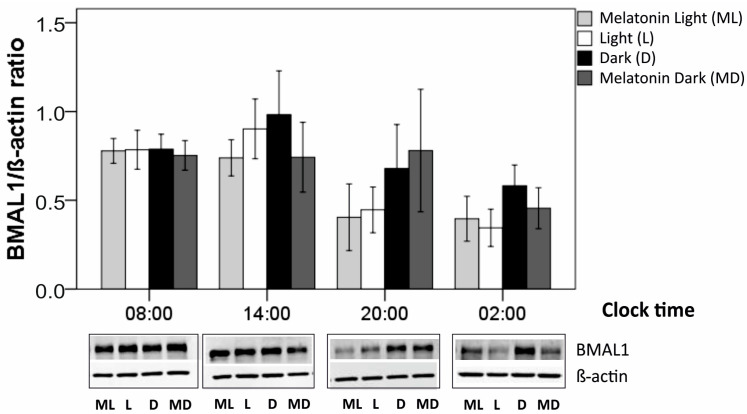
Production of BMAL1 protein in darkness (D), daylight (L), melatonin in darkness (MD), or combined daylight/melatonin (ML) induction (*N* = 5 for each time point and condition). Mean ± SEM are given. Beneath each time point, exemplary blots are shown for BMAL1 and the corresponding β-actin. Group [condition] comparisons (continuous lines) were performed for each clock time using the Wilcoxon test. Clock-time comparisons within each condition (broken line) were done using the Mann-Whitney U test. *, *p* < 0.050.

Regarding clock-time comparisons, in cultured RPE cells, BMAL1 protein production was analyzed at four time-points (08:00, 14:00, 20:00, 02:00) for four different conditions (darkness, daylight, darkness plus melatonin, daylight plus melatonin). Within each condition, BMAL1 production did not differ significantly along the four clock times as a whole time period (each, *p* > 0.050, Kruskal-Wallis tests, Table 2). When comparing only two time points, there was one single significant difference: after light induction, BMAL1 production was higher at 08:00 than at 02:00 (*p* = 0.032, U test, Figure 5). Comparing conditions (Table 2, Figure 5), compared to darkness (VD), light induction (VH) resulted in a decrease in BMAL1 production at most clock time points, being statistically significant at time point 02:00 (*p* = 0.043, Wilcoxon test). By contrast, melatonin induction did not alter BMAL1 production at any clock time point (VD versus MD, VH versus MH, each *p* > 0.050). Combined light/melatonin induction (MH), compared with dark condition (VD), reduced BMAL1 production at all four time points, being significant at time point 02:00 (*p* = 0.043, Wilcoxon test) and 20:00 (*p* = 0.043).

## 4. Discussion

RPE-derived VEGF is of high importance in retinal physiology and is regulated at many levels [6,19]. In this study, we investigated the release of VEGF from RPE cells in vitro in relation to clock time, light, and melatonin. As an in vitro system, we employed single-eye primary porcine cultures. While the use of primary porcine RPE cells is a well-established model [52], the culture of single-eye RPE cells, cultivated in darkness, was established for this study in order to reduce confounding factors, such as the mixture of cells from different donor organisms or irrelevant light cues.

In our RPE cell culture, we found a significant 24 h variation but no circadian rhythm (as determined by cosinor analysis), with a reduction of VEGF over 24 h (12:00, first day – 12:00, second day). The removal of the supernatant may interfere with the expression and secretion of VEGF. As we have shown before, VEGF may be regulated by a positive feedback loop via the VEGF Receptor-2 [13,14]. A repetitive removal of the supernatant and its VEGF over the course of 24 h may reduce VEGF expression by interfering with VEGF-VEGFR-2 interaction. Nevertheless, we found a circadian variation with increased secretion at 24:00, as seen in dark control and daylight-treated samples. In room-light-treated samples, however, the increase at 24:00 was lost, indicating at least some influence of light in the circadian variation of VEGF secretion.

Still, light seems to have little influence on VEGF secretion, as no significant changes of VEGF secretion could be found after light induction. In the literature, a light-induced increase in VEGF has been described for RPE cells, especially in combination with light-induced retinal damage. In light-induced damage models, however, the duration of light exposure massively exceeds the duration used in this study, ranging from 1 h to 24 h in contrast to 20 min used in this study [47,53,54]. In contrast, previous studies have shown that illumination times up to 30 min did not display any toxicity on primary RPE cells [47]. Of interest, even though light exposure did not exhibit an effect on VEGF on its own, the combination of daytime light stimulus with nighttime melatonin had the strongest effect on VEGF content, indicating a preference of VEGF secretion for a more natural environment.

Melatonin in the retina is released by photoreceptors in a circadian fashion, with high concentrations at night and low concentrations during the day [28]. Mimicking this by providing melatonin to our cultures from 20:00 – 8:00, we found that it increased VEGF content in the supernatant of our RPE cells. This seems to be in contrast to previously published studies, where melatonin was shown to reduce VEGF secretion under hypoxia, retinopathy of prematurity, high glucose stimulation, and in the diabetic retina [43,48,55,56]. However, in all of these models, the effect of melatonin on pathologically increased VEGF was measured. In our study, we investigated the constitutive secretion of VEGF. It is known that physiological and pathological VEGF are regulated via different pathways [15,17,18,19]. Indeed, melatonin has been shown to reduce pathological VEGF secretion by inhibiting HIF-1 activation [55], while constitutive VEGF secretion in the RPE is not regulated via HIF-1 [14]. Moreover, melatonin is a known anti-oxidant, protecting the retina and RPE from oxidative insults [41,57], and hypoxia as well as hyperglycemia induce oxidative stress in the retina [58,59,60]. Oxidative stress, on the other hand, is a known inducer of VEGF expression [17,61]. Therefore, the inhibiting effect of melatonin on VEGF secretion in the retina may be associated with pathological VEGF expression only; moreover, it may be related to its anti-oxidant properties. It has to be kept in mind that VEGF is a natural occurring cytokine in the retina with many protective functions, such as keeping up the fenestration of the endothelia of the choroid and protecting endothelial cells, RPE, and photoreceptors from oxidative stress [3,4,10,11,62,63]. Indeed, the prolonged inhibition of VEGF has been implicated in anatomic alterations in the RPE and choroid, in the loss of photoreceptors and visual function in mouse models, and has been discussed as an accelerator of geographic atrophy in AMD [12,64,65,66,67]. Our results indicate a role of nocturnal melatonin (possibly in collaboration with morning daylight exposure) in physiological VEGF secretion of the RPE, possibly connecting it to the protective VEGF functions.

BMAL1 is considered an important factor in the circadian rhythm of the retina [52]. We could see a reduction of BMAL1 protein expression under light conditions at nighttime, but not daytime, fine tuning the expression of clock proteins. This is an interesting finding, as it again stresses the importance of a lifestyle adjusted to the natural surroundings in order to maintain the function of the retina healthy. Light has been shown to influence BMAL1 expression in the retina in some model systems [68] but not in others [51]. However, similar to [51], light did not induce a phase shift in our study but changed the amount of expressed protein. Similarly, in a model using RPE/choroid explants from mice, it was shown that a gentle light impulse of 10 minutes did not show any influence on the inherent RPE circadian rhythm. The influence on BMAL1 expression, however, was not tested in that model [25].

## 5. Conclusions

Our data indicate a circadian variation in VEGF secretion and an influence of nocturnal melatonin on VEGF expression, which can be further increased by morning daylight. This strongly suggests that environmental cues connected to the circadian synchronizing of the retina influence VEGF secretion, adding another layer of complexity to VEGF regulation and indicating the importance of a more natural circadian lifestyle for a healthy retina. This is of even higher interest, as the development of AMD has been thought to be correlated with a reduced responsiveness of retinal tissue to melatonin and a dysregulation of melatonin expression [69,70]. Furthermore, our study shows the importance of distinguishing between constitutive (physiological) and pathologically induced VEGF regulation.

## Figures and Tables

**Table 1:** Variation of VEGF protein content in the supernatant of primary single-eye RPE cultures over 24 h (12:00 to 12:00) under different conditions (darkness, after external light and melatonin intervention conditions).

Condition	*N*	24h-Clock Time Comparison Chi^2^	24h-Clock Time Comparison P^a^
Dark control	40	102.99	5.9 × 10^−20^
Daylight morning (7:40–8:00)	8	24.21	4.8 × 10^−4^
Room light morning (7:40–8:00)	18	43.07	1.3 × 10^−7^
Daylight evening (19:40–20:00)	5	21.26	**0.002**
Room light evening (19:40–20:00)	5	22.97	**0.001**
Melatonin dark	19	23.89	**0.001**
Melatonin daylight (7:40–8:00)	5	19.20	**0.004**
Melatonin room light (7:40–8:00)	14	17.85	**0.007**

^a^ Friedman test. Significant *p* values are depicted in bold.

**Table 2:** Production of BMAL1 protein in single-eye primary RPE cells at different time-points in darkness as well as under external light and melatonin intervention conditions. Protein content was analysed using Western blot, densitometrically quantified, and related to ß-actin control.

Clock Time (hrs)	Ratio BMAL1/ β actin	MH^b^	VH^b^	VD^b^	MD^b^	Chi^2^	P^a^
8:00	N	5	5	5	5		
	Mean	0.778	0.785	0.788	0.753	0.36	0.948
	SEM	0.070	0.109	0.084	0.083		
14:00	N	5	5	5	5		
	Mean	0.739	0.902	0.983	0.743	2.04	0.564
	SEM	0.102	0.168	0-246	0.197		
20.00	N	5	5	5	5		
	Mean	0.404	0.446	0.677	0.780	0.28	**0.041**
	SEM	0.188	0.129	0.248	0.345		
02.00	N	5	5	5	5		
	Mean	0.396	0.345	0.582	0.455	9.00	**0.029**
	SEM	0.126	0.105	0.117	0.115		

^a^ Friedman test. ^b^ No significant differences were observed between the four time points within each condition (*MH: Chi^2^* = 6.06, *p* = 0.109; *VH: Chi^2^* = 7.39, *p* = 0.060; *VD: Chi^2^* = 2.365, *p* = 0.501; *MD: Chi^2^* = 2.76, *p* = 0.430; Kruskal-Wallis tests for independent groups). Abbreviations: MH = Melatonin and daylight; VH = daylight, VD = darkness, MD = Melatonin in darkness. Significant *p* values are depicted in bold.

## Data Availability

The data presented in this study are available on request from the corresponding author.
